# Ambient air pollution exposure and incidence of cataract surgery: The prospective 3City‐Alienor study

**DOI:** 10.1111/aos.16790

**Published:** 2024-11-11

**Authors:** Laure Gayraud, Marion Mortamais, Cédric Schweitzer, Kees de Hoogh, Audrey Cougnard‐Grégoire, Jean‐François Korobelnik, Marie‐Noelle Delyfer, Marie‐Bénédicte Rougier, Karen Leffondré, Catherine Helmer, Danielle Vienneau, Cécile Delcourt

**Affiliations:** ^1^ Univ. Bordeaux, INSERM, BPH, U1219 Bordeaux France; ^2^ University of Montpellier, INSERM, Institute for Neurosciences of Montpellier (INM) Montpellier France; ^3^ Service d'Ophtalmologie Centre Hospitalier Universitaire de Bordeaux Bordeaux France; ^4^ Swiss Tropical and Public Health Institute Allschwil Switzerland; ^5^ University of Basel Basel Switzerland

**Keywords:** air pollution, cataract, eye disease, fine particulate matter, NO_2_, oxidative stress

## Abstract

**Purpose:**

Cataract, the leading cause of blindness worldwide, is a multifactorial disease involving oxidative stress mechanisms. The aim of our study was to investigate the relationship between air pollution exposure and the incidence of cataract surgery.

**Methods:**

The 3C‐Alienor study is a population‐based cohort of residents of Bordeaux, France, aged 65 years or more, recruited in 1999–2000 and followed every 2–3 years until 2017. Cataract surgery was self‐reported and checked at slit‐lamp by trained professionals. Average air pollution exposure (particulate matter ≤2.5 μm (PM_2.5_), black carbon (BC), nitrogen dioxide (NO_2_)) in the 10 years preceding baseline was estimated at the participants' geocoded residential address, using temporally adjusted land use regression. Associations of 10‐year average air pollution exposure with incidence of cataract were estimated using Cox proportional hazard models adjusted for confounders.

**Results:**

The study included 829 subjects without cataract surgery prior to inclusion; the mean age at inclusion was 72.6 years (standard deviation (SD): 4.2) and 61% were women. The median (Interquartile‐range (IQR)) follow‐up duration was 14.1 (6.4) years during which 507 participants underwent cataract surgery. Exposure to a concentration ≥40 μg/m^3^ of NO_2_ (the current regulatory limit value in Europe) was associated with incident cataract surgery (HR = 1.46, CI (1.16, 1.84), *p* = 0.001). No statistically significant association was found with PM_2.5_ and BC.

**Conclusion:**

Long‐term exposure to a NO_2_ concentration ≥ 40 μg/m^3^ was associated with an increased incidence of cataract surgery. Complying with current European air pollution standards could reduce cataract surgery costs and improve population quality of life.

## 1 INTRODUCTION

Cataract is defined as the progressive opacification of the lens and represents the first cause of blindness worldwide (GBD 2019 Blindness and Vision Impairment Collaborators & Vision Loss Expert Group of the global burden of disease study, 2021). This opacification is characterized by a gradual loss of visual acuity and contrast perception leading to impaired quality of life (Mencucci et al., 2023). The prevalence of cataract increases with age, from 4% at age 60 to 93% at age 80 and older (Cicinelli et al., 2023; Liu et al., 2017). As the population ages, the number of cases tends to increase, representing a considerable burden on healthcare systems and the economy worldwide. The only effective treatment is surgery, which requires access to specialized equipment and skilled surgeons that are not available in all countries or regions and represents a major cost in many countries. In the United States, the cost of cataract surgery is estimated at $3.4 billion per year (Fang et al., 2022).

The mechanisms underlying the development of cataract have not yet been clearly identified; however, oxidative stress is a well‐established factor (Johra et al., 2020; Ottonello et al., 2000; Selin et al., 2014). Nitrogen dioxide and fine particulate matter (PM_2.5_ (particular matter with diameter ≤ 2.5 μm), black carbon (BC)) are air pollutants mainly produced by human combustion activities (Hoffmann et al., 2021). Chronic exposure to air pollution can exacerbate Reactive Oxygen Species (ROS) production and may overwhelm the natural antioxidant defences of the lens. Indeed, the lens is rich in proteins that can be modified by ROS‐induced oxidation, leading to protein aggregation and cross‐linking, resulting in lens opacity. These mechanisms have been confirmed in animal experiments (Balasubramanian et al., 1990; Kubo et al., 2018; Ottonello et al., 2000). To date, only four epidemiological studies, of which only two were longitudinal, have investigated the relationship between air pollution exposure and cataract in Canada, the United Kingdom and South Korea (Choi et al., 2018; Chua et al., 2021; Shin et al., 2020), and their findings are inconclusive. In the two large longitudinal cohorts in the United Kingdom and South Korea, exposure to high NO_2_ levels was associated with a higher risk of cataract surgery, but not with cataract diagnosis in a South Korean cross‐sectional study (Choi et al., 2018; Chua et al., 2021; Shin et al., 2020). The results were even less consistent regarding fine particulate matter, with an increased risk of cataract surgery in England for participants with higher exposure to PM_2.5_ (Chua et al., 2021), but not in the Canadian cross‐sectional study (Grant et al., 2021) or the South Korean longitudinal study (Shin et al., 2020). Moreover, these studies had significant limitations. The exposure measurements were imprecise; NO_2_ estimations for 2010 were used for the entire study period in the United Kingdom (Chua et al., 2021), while South Korean studies relied on the nearest measurement station without extrapolation (Choi et al., 2018; Shin et al., 2020). Additionally, in Canada, cataract surgery was self‐reported without verification (Grant et al., 2021). Moreover, the concentration ranges were much higher in South Korean studies (Choi et al., 2018; Shin et al., 2020) and significantly lower in the United Kingdom and Canada (Chua et al., 2021; Grant et al., 2021), lacking information on the effects of medium‐range exposure levels, as observed in European cities. Finally, none of the previous studies adjusted for ultraviolet exposure, a well‐known factor associated with the development of cataracts and implicated in the formation of air pollutants.

Thus, our study aimed at further investigating the relationship of exposure to air pollutants (NO_2_ and fine particulate matter, including black carbon (BC)) with the incidence of cataract surgery in a prospective French cohort over an 18‐year follow‐up.

## 2 MATERIALS AND METHODS

### 2.1 Setting and study population

**FIGURE 1:**
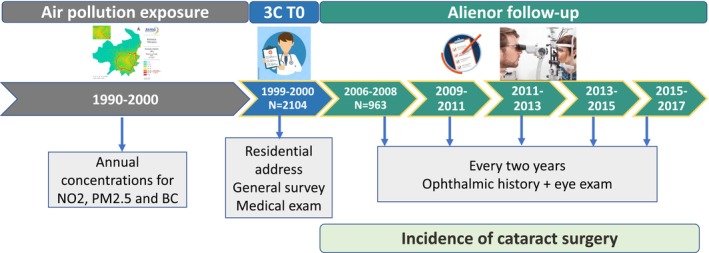
Study design.

The Three‐City (3C) study is an ongoing prospective population‐based cohort study on the vascular risk factors for dementia, including 9294 subjects registered on the electoral list from three French Cities (Bordeaux *n* = 2104, Dijon *n* = 4931 and Montpellier *n* = 2259) (3C Study Group, 2003). Participants over 65 years of age were recruited in 1999–2000 and followed every 2 years (Figure 1). Among the 2104 individuals in the 3C Bordeaux cohort, 963 were included in the Alienor cohort.

From 2006 onwards (the third 3C follow‐up), an ophthalmological check‐up was offered every 2 years to all participants of 3C Bordeaux still alive, as part of the Alienor (Antioxydants, Lipides Essentiels, Nutrition et maladies OculaiRes) study, which aimed at assessing the association of age‐related eye diseases with nutritional, environmental and genetic factors (Delcourt et al., 2010). Currently, the ophthalmological follow‐up is available until 2017 (six visits) for 963 subjects who agreed to participate.

This research was approved by the Ethical Committee of Bordeaux (Comité de Protection des Personnes Sud‐Ouest et Outre‐Mer III) for Alienor and from the Ethical Committee of the University Hospital of Kremlin‐Bicêtre for 3C and followed the declaration of Helsinki's tenets. All participants provided informed written consent for enrolment in the study.

### 2.2 Cataract surgery assessment

Cataract surgery was reported by participants and checked at slit lamp at each eye examination. The year of cataract surgery was defined as the self‐reported year of first cataract surgery. Incidence of cataract surgery was defined as first cataract surgery performed between the baseline 3C (1999–2000) and the fifth Alienor visit (2017), in participants without cataract surgery at baseline.

### 2.3 Air pollutant exposure assessment

Air pollution maps were developed as part of the ELAPSE (Effects of Low‐Level Air Pollution: A Study in Europe) study for Western Europe representing annual average concentrations of particulate matter (PM_2.5_ (μg/m^3^), black carbon (10^−5^/m) and NO_2_ (μg/m^3^)). They were based on 100 × 100 m land use regression models (LUR) which take into account measurements recorded in 2010 at a respective 2399 and 543 NO_2_ and PM_2.5_ air quality monitoring stations from the European Environment Agency Airbase network, and 436 purposely sampled locations for BC (Eeftens et al., 2012), and predictor variables related to land use characteristics, distance to roads, altitude and distance to the sea, population density, chemical transport model and satellite‐derived measurements (de Hoogh et al., 2018, 2013; Eeftens et al., 2012). The models explained 66%, 51% and 58% of the spatial variation of the measured PM_2.5_, BC and NO_2_ concentrations in model building, respectively. Using temporal air pollution data and regional boundaries, the 2010 model estimates were extrapolated for the 1990–2016 period. Thus, annual air pollution exposure was estimated from 100 × 100 m LUR models, at the geocoded residential address of the participants. For each subject and pollutant, exposure was estimated and assigned at baseline as the 10‐year average prior to the 3C baseline (Figure 1).

### 2.4 Covariates

Data were collected every 2 years at each 3C follow‐up during a face‐to‐face interviews using standardized questionnaire administered by a trained psychologist or nurse. The covariates were selected based on literature and were taken into account at baseline. They included socio‐demographic variables such as age, gender, income and deprivation index, an indicator of the contextual socio‐economic status of each participant at the neighbourhood level in tertiles (Letellier et al., 2018). Comorbidities included diabetes (fasting blood glucose ≥7.0 mmol/L or non‐fasting blood glucose ≥11 mmol/L or use of antidiabetic medication), hypertension (systolic blood pressure ≥ 140 mm Hg and/or diastolic blood pressure ≥ 90 mm Hg and/or use of antihypertensive drugs), asthma (self‐reported) for its association with corticosteroids use, and oral corticosteroid therapy (self‐reported). The Body Mass Index (weight (kg)/height (m)^2^) was presented in three categories (<25, [25–30], >30, as defined by the WHO), as well as smoking status (never smoking, <20 pack‐years, ≥20 pack‐years). Lifetime exposure to ambient total ultraviolet radiation (UVR) was estimated using residential history and Eurosun satellite‐based estimations of ground UVR (for more details see Delcourt et al., 2014) and divided into quartiles.

### 2.5 Statistical analysis

First, the population included in this analysis was compared to the whole population of 3C Bordeaux regarding socio‐economic status, lifestyle, comorbidities and air pollution exposure.

The survival curves were compared using Kaplan–Meier estimation and log‐rank tests regarding the average exposure to pollutants in the 10 years prior to baseline.

Finally, the relationships between NO_2_, PM_2.5_ and BC exposure and cataract surgery incidence were assessed using Cox proportional hazard models. The time axis was the time elapsed from baseline to the first eye to undergo cataract surgery or to the last patient's visit. All models were single pollutant models due to strong inter‐correlations of the pollutants (Pearson correlation coefficient > 0.7).

Two models were selected based on potential confounders identified in literature. The main model (model one) was adjusted for age, gender and social deprivation index. The model two was further adjusted for diabetes, smoking status, asthma, hypertension, BMI and ambient UVR (treatment with corticosteroids was not taken into account due to the small number of subjects) after imputation of missing covariate (percentage of missing values: diabetes (7.5%), smoking status (1.3%), BMI (0.8%), UVR (13.9%)) data using MICE package (van Buuren & Groothuis‐Oudshoorn, 2011).

The linearity of quantitative variables was checked using 3‐node restricted cubic splines, and the proportionality of hazards was assessed with the Schoenfeld test. Since linearity was not respected for NO_2_, this variable was dichotomized at 40 μg/m^3^, corresponding to the European regulatory limit. Additionally, as age did not respect the proportionality of risks, we dichotomized age‐related hazard into two periods: before and after 10 years of follow‐up. All statistical analyses were performed using R, version 4.0.3 (R Core Team) with the package Survival.

## 3 RESULTS

### 3.1 Descriptive analysis

**FIGURE 2:**
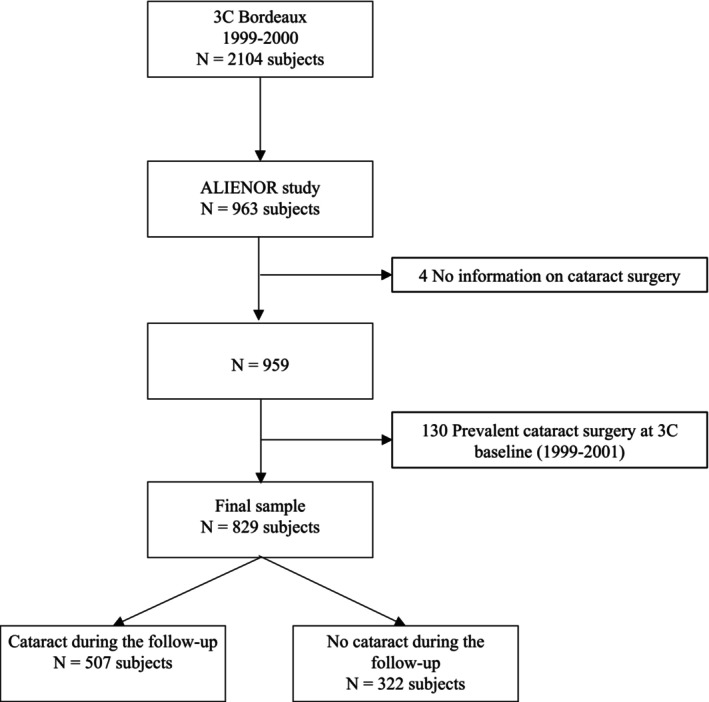
Flow chart.

Of the 2104 participants in the 3C Bordeaux cohort, 963 were enrolled in the Alienor cohort. Four participants had missing data concerning cataract surgery and 130 subjects had undergone cataract surgery prior to inclusion in 3C and were therefore excluded (Figure 2). Among the 829 included participants, 61% were females and the mean age was 72.6 years (standard deviation (SD): 4.2) (Table 1). Overall, 55% of the sample had an income between €1000 and €3200 per month, two‐thirds had never smoked and 17% had a BMI of 30 kg/m^2^ or more. A large proportion of participants were hypertensive (76%), less than 10% had diabetes and 8% had asthma. The participants were exposed to an average ambient UVR exposure of 40.2 KJ/cm^2^ (SD: 2.0). Compared with the total 3C Bordeaux cohort (*N* = 2104), the study sample (*n* = 963) tended to be younger at baseline, with an average age of 72.6 years compared to 74.6 years, slightly more often in the upper income range, and had more often diabetes (12% vs. 9%) (Table 1). Nonetheless, for the remaining characteristics, no substantial differences were observed.

**TABLE 1:** Characteristics of the included population and the 3C Bordeaux population at baseline (1999–2000).

Baseline characteristics	3C Bordeaux, *N* = 2104	Included population, *N* = 829
	*N* (%)	*N* (%)
Female	1288 (61)	503 (61)
Age, Mean (SD), year	74.64 (5.10)	72.57 (4.19)
Income (Euros)	*N* = 2074	*N* = 823
<1000	208 (10)	59 (7)
≥1000 and <3200	1194 (58)	455 (55)
≥3200	540 (26)	262 (32)
Does not wish to answer	162 (6)	53 (6)
Deprivation index	*N* = 2052	*N* = 815
Low	739 (36)	298 (37)
Medium	666 (32)	260 (32)
High	647 (32)	257 (32)
Smoking (pack‐years)	*N* = 2070	*N* = 818
Never smoker	1354 (65)	529 (65)
<20	336 (16)	151 (18)
≥20	380 (18)	138 (17)
Oral corticosteroid therapy	37 (2)	8 (1)
Body Mass Index, kg/m^2^	*N* = 2048	*N* = 822
<25	818 (40)	310 (38)
≥25 and <30	886 (43)	376 (46)
≥30	344 (17)	136 (17)
Hypertension	1661 (79)	626 (76)
Diabetes	*N* = 1874	*N* = 767
	221 (12)	66 (9)
Asthma	164 (8)	63 (8)
Lifetime ambient UVR (KJ/cm^2^)	*N* = 807	*N* = 714
	40.2 (2.0)	40.2 (2.0)
NO_2_, μg/m^3^ (Median (IQR) Min–Max)	34.29 (7.10) 25.41–73.25	34.13 (7.52) 25.57–73.25
PM_2.5_, μg/m^3^ (Median (IQR) Min–Max)	30.01 (1.29) 22.84–34.17	30.02 (1.25) 22.84–34.17
BC, 10^−5^/m (Median (IQR) Min–‐Max)	2.41 (0.31) 1.83–3.89	2.42 (0.32) 1.83–3.89

Abbreviations: BC, black carbon; IQR, interquartile range; KJ, kilojoules; Max, maximum; Min, minimum; NO_2_, nitrogen dioxide; PM2.5, fine particulate matter ≤2.5 μm; SD, standard deviation; UVR, ultraviolet radiation.

The median follow‐up duration was 14.1 years (Min–Max [6.5–17.9]), during which 507 participants underwent cataract surgery.

Regarding exposure to air pollution, the median (interquartile range (IQR)) of the sample was 34.13 μg/m^3^ (7.52) for NO_2_, 30.02 μg/m^3^ (1.25) for PM_2.5_ and 2.42 10^−5^/m (0.32) for BC.

### 3.2 Univariate and multivariate analyses

**FIGURE 3:**
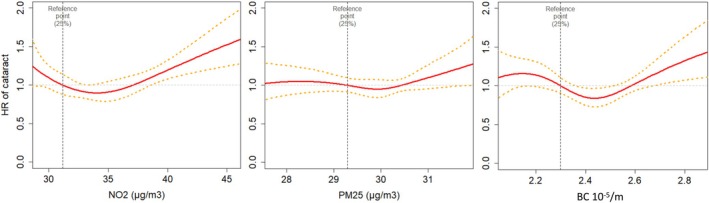
Associations of risk of cataract surgery with air pollutants (3C‐Alienor study, 1999–2017), estimated using cubic splines.

Figure 3 illustrates the association of cataract surgery risk with varying concentration levels of air pollutants, using Cox modelling with cubic splines, adjusting for age, gender and social deprivation index. The relationship of NO_2_ exposure with incident cataract surgery was non‐linear. The hazard ratio for cataract surgery significantly increased for NO_2_ concentrations above 40 μg/m^3^, which corresponds to the current EU regulatory limit. We thus categorized NO_2_ exposure in two categories (above and below 40 μg/m^3^). No effect was observed below this threshold, and no effect was observed with PM_2.5_ and BC (Figure 3).

**FIGURE 4:**
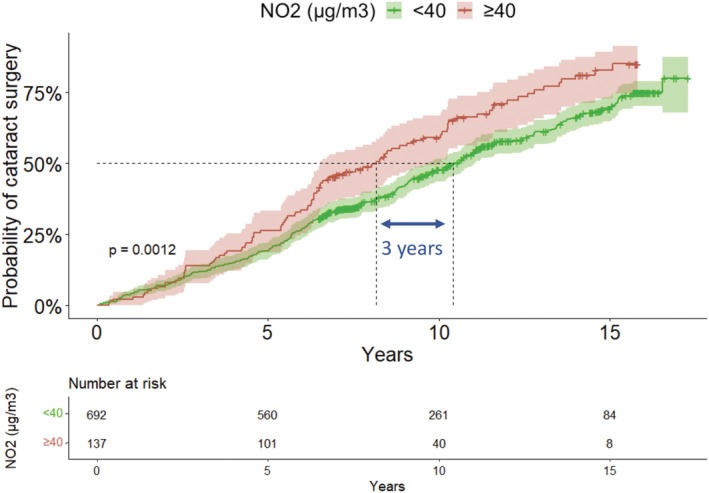
Probability curves of cataract surgery with NO_2_.

Figure 4 shows the probability curve of cataract surgery comparing NO_2_ exposure above and below 40 μg/m^3^. From 6 years of follow‐up onwards, participants with NO_2_ exposure above 40 μg/m^3^ reached the same probability of cataract surgery almost 3 years earlier than those exposed to less than 40 μg/m^3^ of NO_2_.

The results of the Cox model fits are presented in Table 2. NO_2_ exposure greater than 40 μg/m^3^ was associated with an increased incidence of cataract surgery (HR = 1.46 (95% confidence interval (CI), 1.16–1.84, *p* = 0.001) after adjustment for age, sex, deprivation index, diabetes, asthma, hypertension, lifetime ambient UVR exposure, BMI and smoking status (Model 2). These results remained consistent and significant in the two adjustment sets. There was no significant association with PM_2.5_ or BC (HR = 1.03 (95% confidence interval (CI), 0.95–1.11), per IQR, *p* = 0.4 and HR = 1.16 (95% confidence interval (CI), 0.78–1.72), per IQR, *p* = 0.55 respectively) (Model 2).

**TABLE 2:** Hazard ratios (95% CI) for the association between air pollutants and cataract surgery risk during the follow‐up (3C‐Alienor study, 1999–2017).

Pollutant exposures	Model 1 *N* = 829			Model 2 *N* = 829		
	HR	95% CI	*p*‐value	HR	95% CI	*p*‐value
NO_2_ (μg/m^3^)						
≥40 versus <40	1.37	1.09, 1.72	0.006	1.46	1.16, 1.84	0.001
PM_2.5_ (μg/m^3^)	1.04^a^	0.95, 1.15	0.38	1.03^a^	0.95, 1.11	0.40
BC (10^−5^/m)	1.05^a^	0.93, 1.19	0.43	1.16^a^	0.78, 1.72	0.55

*Note*: Model 1, adjusted for age, sex and deprivation index; Model 2, further adjusted for diabetes, asthma, hypertension, lifetime ambient UVR exposure, BMI and smoking status after multiple imputation of the missing data for covariates.

Abbreviations: CI, confidence interval; HR, hazard ratio.

^a^ Per IQR (PM_2.5_ = 1.25 μg/m^3^, BC = 0.32 10^−5^/m).

## 4 DISCUSSION

In this cohort of older individuals from Bordeaux, exposure to a 10‐year average of NO_2_ concentrations above 40 μg/m^3^ at baseline was associated with an approximately 50% increased risk of undergoing cataract surgery by comparison with an exposure below 40 μg/m^3^. No association was found with exposure to fine particulate matter (PM_2.5_ and BC).

These results are consistent with those found in the literature (Grant et al., 2022). Indeed, NO_2_ is the pollutant most often associated with an increased risk of cataract in the previous studies. To date, only three national studies, including one in the United Kingdom and two in South Korea, have investigated the relationship between exposure to NO_2_ and cataract (Choi et al., 2018; Chua et al., 2021; Shin et al., 2020). Of these, two found an increased risk of cataract surgery for participants with higher NO_2_ exposure. Among 433 000 participants in the UK Biobank, those in the highest quartile of NO_2_ exposure (Q4: 33.86 to 125.12 μg/m^3^) were 11% more likely to undergo cataract surgery compared to the lowest quartile (Q1: 8.86 to 22.91 μg/m^3^) (Chua et al., 2021). In 115 000 South Koreans, the hazard ratio (HR) was 1.080 (95% confidence interval 1.030–1.133) for those exposed to the highest quartile (Q4: 26.1 to 29.1 ppb vs. Q1: 23.0 to 24.0 ppb) of NO_2_ (Shin et al., 2020). In these two studies, the HR were substantially lower than in the present study; these differences might be due to the lower cut‐offs for the high‐exposure groups, the shorter follow‐up duration and the younger age of the participants.

No association with NO_2_ exposure was found in a second South Korean study. However, this study had some limitations (Choi et al., 2018). Indeed, it was cross‐sectional. Moreover, pollution levels were estimated at the district level, which is a much cruder measure compared to residential address assessments. The estimated concentrations were about twice as high as those observed in our sample and, in general, significantly higher than those typically seen in Europe, making direct comparisons challenging. Additionally, this study relied on self‐reported cataract diagnoses rather than surgical interventions. Cataracts are a natural ageing process of the eye that can develop very slowly before surgery becomes necessary. Therefore, undergoing surgery is indicative of a more advanced stage of cataract.

We did not find any association with exposure to fine particulate matter (PM_2.5_ and BC). This finding is consistent with the results of a cross‐sectional study involving 30 000 Canadian participants and a South Korean longitudinal study (Grant et al., 2021; Shin et al., 2020). However, among participants from the UK Biobank, those exposed to PM_2.5_ in the highest quartile (Q4: 10.57 to 21.31 μg/m^3^) were found to be 14% more likely to undergo cataract surgery compared to those in the lowest quartile (Q1: 8.17 to 9.29 μg/m^3^) (Chua et al., 2021). This difference may be attributed to differences in exposure ranges. Indeed, the range of exposure to PM_2.5_ in the United Kingdom (estimated for year 2010) was between 8 and 21 μg/m^3^, compared to 23 to 34 μg/m^3^ in our study. Given the dissimilar concentration ranges, an effect at lower thresholds with a ceiling effect at higher concentrations cannot be ruled out. In addition, the composition of fine particulate matter might also differ between study areas. PM_2.5_ and BC consist of a mixture of various constituents, some of which are more toxic, such as heavy metals. Moreover, the follow‐up periods and durations were different, as well as the age of participants. Additionally, access to healthcare may differ on a national scale, such as in the United Kingdom, compared to more localized areas with a good supply of healthcare professionals, like the Bordeaux metropolitan area.

The biological mechanisms underlying the effect of pollutants on eye diseases have been confirmed by experimental studies in animals (Kang et al., 2020; Quan et al., 2021). It is well established that pollution increases the levels of reactive oxygen species (ROS) and that oxidative stress is an underlying mechanism in the development of cataracts (Lodovici & Bigagli, 2011). Indeed, the lens is rich in proteins, especially crystallins, which are essential for maintaining lens clarity. ROS can alter these lens proteins through oxidation, causing protein aggregation and cross‐linking. These changes compromise the solubility and functionality of crystallins, leading to lens opacity. Additionally, the lens contains membrane lipids susceptible to peroxidation in the presence of ROS. Lipid peroxides can then further react with lens proteins, intensifying protein damage and aggregation. This mechanism is also a contributing factor to cataract formation. Furthermore, ROS can induce oxidative damage to DNA. This can impair the ability of lens epithelial cells to repair and replicate, potentially leading to cell death and further structural disorganization (Hsueh et al., 2022; Kubo et al., 2018; Ottonello et al., 2000; Quan et al., 2021).

The strengths of our study include its longitudinal design, with participant follow‐up extending over 18 years. The robustness of the estimations was enhanced by using Land Use Regression (LUR), along with an extrapolation that accounted for meteorological and geographical factors, which allowed for exploring a long‐term exposure period based on the residential addresses. Lifetime UVR exposure, a well‐known risk factor for cataracts (Delcourt et al., 2014), was also taken into account, along with all major cataract risk factors (smoking, diabetes, asthma, hypertension, etc.). Additionally, the confirmation of cataract surgery was checked by trained professionals.

Our study has some limitations. Indeed, the date of the cataract surgery was self‐reported. However, this information was collected consistently throughout the follow‐up period, which limits recall bias. It is possible that some participants needed but did not receive cataract surgery. Nevertheless, in France, cataract surgery is fully covered financially by the national health insurance system, and our analysis revealed no differences in cataract surgery based on socio‐economic status (data not shown). Moreover, ophthalmologists were easily accessible to the entire population, as the metropole of Bordeaux is well endowed with healthcare professionals.

The lack of a residential history limited our observation of the effect of pollution and did not allow us to explore different exposure windows or to consider the air pollution exposure over time since the inclusion. Given that cataract development is a slow process, it is more likely that long‐term rather than short‐term air pollution exposure affects its progression. Therefore, we chose to consider the average exposure over the 10 years preceding inclusion in the study, despite having residential information only at the 3C study's baseline. This decision was based on data from the French National Institute of Statistics, which indicated that around the year 2000, the relocation rate among the population over 60 years old in the Bordeaux region was very low. Finally, the limited size of our sample and the restricted geographical area resulted in a narrow range of exposure. This might have led to a low statistical power, potentially explaining the lack of association found with fine particulate matter.

## 5 CONCLUSION

In conclusion, this study suggests an almost 50% increase in the risk of cataract surgery in individuals exposed to NO_2_ levels exceeding 40 μg/m^3^. This concentration represents the current regulatory threshold of the European Union; however, it is still not achieved in several cities across Europe. Adhering to this threshold could likely lead to a reduction in medical expenses for society. Indeed, cataract surgery constitutes a significant financial burden worldwide, a situation exacerbated by an ageing population.

## FUNDING INFORMATION

Théa Pharma participated in the design of the Alienor study, but none of the sponsors participated in the collection, management, statistical analysis and interpretation of the data, or in the preparation, review or approval of the present manuscript.

## Data Availability

The dataset presented in this article are not readily available because of ethical and legal restrictions. Requests to access the dataset should be directed to the Steering Committee of the Alienor study (contact corresponding author).
